# Human *in vitro* models of neurovasculature and the application to pre-clinical intracerebral haemorrhage research

**DOI:** 10.1016/j.bioactmat.2025.10.018

**Published:** 2025-10-25

**Authors:** Siobhan Crilly, Mihai Lomora

**Affiliations:** a School of Biological and Chemical Sciences, College of Science and Engineering, University of Galway, Galway, Ireland; b CÚRAM, Research Ireland Centre for Medical Devices, University of Galway, Galway, Ireland; c Institute for Health Discovery and Innovation, University of Galway, Galway, Ireland

**Keywords:** Transwell, Biomaterials, Microfluidics, Brain organ-on-chip, Intracerebral haemorrhage

## Abstract

Haemorrhagic stroke continues to be a leading cause of death and disability globally, with limited treatment options. Pre-clinical models must adapt to offer translationally relevant and physiologically accurate alternatives to animals. The development of complex co-culture blood-brain barrier models and the incorporation of hydrogels and biomaterials has resulted in microphysiological 3D platforms. Amongst such 3D platforms, cerebral organoids have transformed the field of neuroscience. Additionally, emerging techniques for vascularisation and perfusion now provide, for the first time, an entirely human-based model of the cerebrovasculature. In this review, we explore the relevance of various *in vitro* platforms - such as transwells, hydrogels and other biomaterials, microfluidics, spheroids, organoids, and perfusion-based systems - for pre-clinical research into haemorrhagic stroke. We discuss the advantages and limitations of each model, with a particular focus on the aspects of disease pathophysiology that hold promise for clinical translation. With some adaptation, intracerebral haemorrhage research can benefit from these models for elucidating pathology and recovery mechanisms that can be exploited therapeutically.

## Highlights

• This review underlines the urgency for a fully human *in vitro* model of intracerebral haemorrhage (ICH) pathology.
• An in-depth insight into the advantages and limitations of 2D and 3D in vitro model systems for ICH disease modelling.
• Recommendations for the adaptation of microphysiological 3D brain models for comprehensive ICH modelling.

## Graphical abstract

**Figure:**
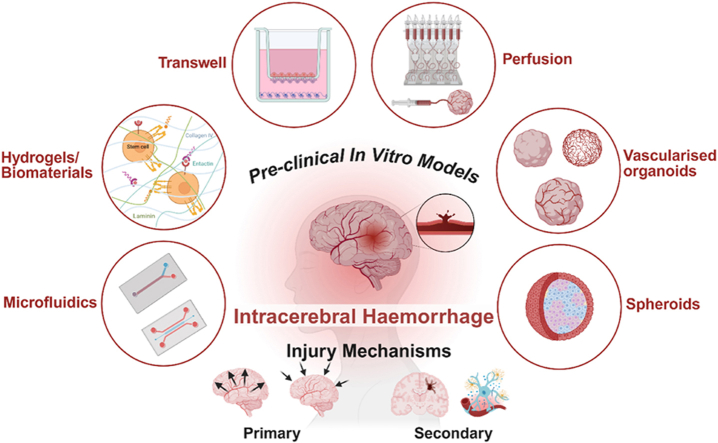
Created in BioRender. Lomora, M. (2025) https://BioRender.com/v8poxfs.

## 1 Introduction

Pre-clinical research into intracerebral haemorrhage (ICH) mechanisms, pathology and therapy is mostly limited to animal models. In recent years there has been an increase in animal models available for initiating cerebral haemorrhage including in different species [[1], [2], [3]]. A translational gap remains between pre-clinical research and applicable treatments progressing to the clinic, due to incomplete understanding of disease aetiology and therapeutic approach. A significant number of pre-clinical studies use animal-derived cells due to their ease of acquisition and maintenance but with little demonstrable translational relevance. This translational gap means a fully human *in vitro* model with the multi-system complexity of ICH pathology is increasingly desirable, for translational and scalable investigation into therapeutic approaches that compares with advances for ischaemic stroke investigation [4].

### 1.1 ICH pathology mechanisms

Physiologically, when cerebral blood vessels spontaneously rupture, blood is released into the brain parenchyma creating a mass effect and mechanisms of injury have recently been reviewed [5]. Briefly, primary injury is caused by the volume of blood within the skull compressing the surrounding brain structures and increasing intracranial pressure. Loss of circulation to surrounding areas contributes to brain cell death through tissue necrosis, ferroptosis and apoptosis. Secondary inflammatory injury is caused by the contact with toxic blood compounds and breakdown products of red blood cells (e.g., haemoglobin, hemin, iron, thrombin) [6]. Activated microglia within brain tissue release pro-inflammatory cytokines (interleukin (IL)-1) that activates astrocytes (IL-6 and matrix metalloproteinase (MMP)-9) and attracts peripheral immune cells to the brain. The extravasation of these cells further damages the integrity of the blood-brain barrier (BBB) and increases brain oedema and intracranial pressure exacerbating the injury and often causing haematomal expansion. Recovery differs from ischaemic stroke with an initial delay, followed by a steep improvement and then gradual changes beyond 3 months [7]. These pathological aspects are critical to predicting clinical disease severity however, cannot be replicated in current *in vitro* systems [8,9].

### 1.2 Limitations with model systems

The complex, multi-systemic pathology of ICH presents major difficulties when attempting to replicate disease pre-clinically. Current *in vitro* models for ICH research are limited by a combination of basic microphysiological structure, lack of co-culture complexity in three dimensions, and fluidic flow. However, the advancements to 3D culture and microfluidics in organ-on-chip models are promising, albeit with some adaptation [10]. *In vitro* models of pathology are often created by adding blood-breakdown factors in isolation to cell culture media such as hemin and haemoglobin [8] but these models fail to replicate the complex cascade of injury mechanisms [11]. Both *in vitro* and animal model systems often fail to replicate key clinical features of disease such as expansion of the haematoma, spreading depolarisation, white matter injury and blood pressure changes, or underlying co-morbidities such as vascular ageing and associated functional neurological changes. Functional outcomes of ICH are important clinical measures of disease severity, such as lethargy, aphasia, hemiparalysis, psychological disorders, pain and loss of cognition. Such parameters are more accessibly monitored in an animal system and would be largely impossible in *in vitro* systems. Biomechanical influence over brain tissue and how these changes over a lifetime is an important consideration when evaluating repair mechanisms, and changes to gross brain morphology following a loss of circulation. Challenges of modelling a multi-system, multi-phase disease likely results in incremental additional complexity, and consistently addressing these advances is vital.

### 1.3 The future of *in vitro* modelling

**Fig. 1:**
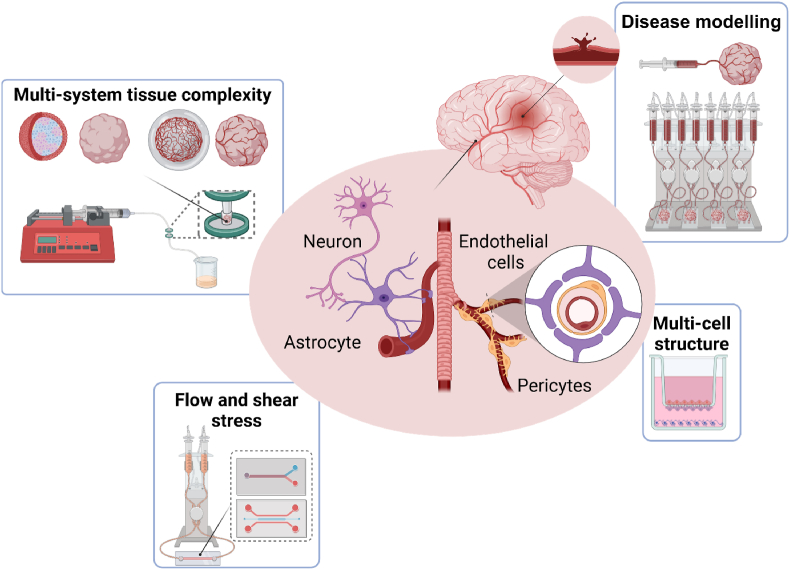
**Complexities of blood-brain barrier physiology and intracerebral haemorrhage pathology and the application of culture systems to address these important model parameters.** To recreate the *in vivo* physiology of the neurovascular unit and the cerebral tissue requires the 3D culture of multiple cell types and maintaining physiological function (e.g. endothelial cell tight junction expression, neural signalling). Pathological mechanisms of intracerebral haemorrhage include aspects of mechanical stress, flow and swelling, and an inflammatory component that is largely overlooked in current organoid cultures. Creating a model to replicate aspects of disease is also required to be high-throughput, reproducible and reliably consistent. Created in BioRender. Lomora, M. (2025) https://BioRender.com/hqamedz.

**Fig. 2:**
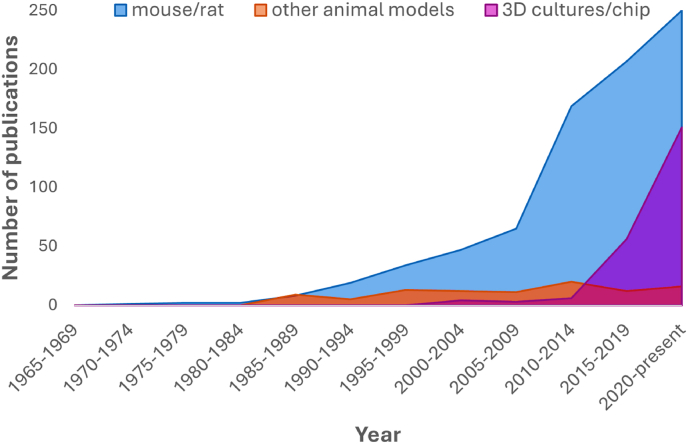
**Trends in publications concerning the neurovascular unit/blood-brain barrier over the past 60 years** reveal a sharp increase in recent years of articles containing terms such as ‘spheroid’, ‘organoid’, ‘3D’ and ‘chip’ in the title. Articles were identified using NCBI search terms “blood-brain barrier”, “neurovascular unit” and “model” until July 2024, associated terms for species (“rat”, “mouse”, “rodent”, “porcine”, “pig”, “zebrafish” etc) and cell-based model system (“*in vitro*”, “chip” “organoid”, “spheroid”) were used for inclusion criteria for each group and plotted against time of publication.

The future generation of ICH models will build on the current state-of-the-art in 3D cerebral organoid culture, using a combination of *i)* both “in house” differentiation protocols and commercially available differentiation kits, *ii)* biomaterials that optimise the extracellular environment for both neural and vascular cells, and *iii)* custom designed microfluidic chips to generate a reproducible and scalable model of complex pathology (Fig. 1). Organoids can be applied to investigating some of the incremental challenges raised above. Guided differentiation results in organoids of specific regional morphology and cell types such as hind brain/basal ganglia and cortex. Incorporating tunable biomaterial matrices with brain specific extracellular matrix (ECM) molecules such as hyaluronic acid and collagen IV, to exert the mechanical forces during expansion and contraction also adds complexity to the microenvironment. Matrices that hold nanoparticles or slow molecular diffusion would provide structured interaction with growth factors such as those that promote neurogenesis (BDNF) and angiogenesis (VEGF) with spatial differentiation. Open cell culture platforms can be used to generate the spontaneous nature of vessel rupture and the critical involvement of the peripheral immune system that contribute to ICH injury in a controlled system. Introducing genetic mutations to mimic co-morbidities such as vascular ageing and pathogenic morphology can reveal molecular associations for targeted therapies. In particular, organoids have the potential to contribute to personalised medicine, sourcing induced pluripotent stem cells or blood/immune cells from patients contributing to the model development. Understanding the contribution of genetic conditions to the spontaneous nature of ICH would offer therapeutic strategies beyond gene therapy and reflect the heterogeneity of the clinical sample. Although organoids are far from being as structurally complicated and organised as the human brain and obviously lack the capacity to infer some functional outcomes of stroke that animals are suited to, the models offer an attractive and more human-relevant intermediate between 2D formats and animal models. Increased complexity of culture system often results in low-throughput experiments, however the drive to increase reproducibility in organoid cultures, and the labour and parts involved in bespoke microfluidic systems, does not lend favour to one model system over the other. The development of these models will provide a translationally relevant alternative to animals, to complement the *in vivo* studies and allow for validation in an appropriate human system. These trends in research are reflected in the increase of studies that reference terms such as ‘spheroids’, ‘organoids, ‘chip’ and ‘3D’, when compared to animal-related terms (Fig. 2). In terms of therapeutics, a small, reproducible, and critically, human platform will allow for applications for drug-development and screening of innovative nanotechnology.

## 2 Traditional *in vitro* models

### 2.1 Application of static 2D models of vascular networks in ICH research

Historically, the models for interrogation of the BBB have been heavily reliant on *in vivo* models (i.e., animal models) due to the structural importance and complexity of the neurovascular unit. However, the high technical skill requirement, time constraints and ethical restrictions posed by *in vivo* models have driven the development of novel *in vitro* models to improve accessibility and translation [12]. The complexity of the cell network that makes up the BBB includes vascular endothelial cells, encircling pericytes, astrocytic endfeet and basement membrane matrix, all of which has tightly regulated structure in the physical system that is challenging to recreate artificially in an accurate manner. Benchmark parameters for characterisation of a human *in vitro* BBB have been outlined to include tight junction expression and transporter proteins [13]. However, BBB models are often validated with assays for cell structure and viability, immunofluorescent analysis of tight junction expression of vascular endothelial (VE)-cadherin and zona occludens (ZO)-1, trans-endothelial electrical resistance (TEER) analysis, morphological changes under shear stress, optical imaging, transmission electron microscopy [14] and genetic profiling of cells. Some attempts have been made to model BBB permeability using deep-learning *in silico* methods incorporating this experimental data [15]. Isolation of brain microvascular cells (hBMVEC or hCMVEC) has allowed for the immortalisation and commercialisation of human brain specific cell lines [16]. Early study models include co-cultures of neuronal cell lines and endothelial cells, or tri-culture models with astrocytes and mixing of cell species origins. Evidence shows that the inclusion of astrocytes in cell monolayers increases physiological relevance, improving tight junction protein expression, enzyme activity, and TEER, and restricts paracellular transport and efflux transport inhibition [17]. Co-culture models with different cell types (astrocytes or pericytes) demonstrate a more accurate representation of the physiological environment and the release of neuronal and angiogenic growth factors [18]. Additional complexity arises from the cell types used in static culture, from immortalised cell lines [19], immune cells, primary cells acquired from animals [20] and human patients of neurological and vascular diseases, differentiated induced pluripotent stem cells (iPSCs) [[21], [22], [23], [24]] to co-culture with animal *ex-vivo* [25] and organotypic brain slices [26,27] and isolated microvessels [28,29]. These models have been very useful for nutrient [30] and drug [[31], [32], [33], [34]] transcytosis, viral infection [35] and nanoparticle absorption [36,37] studies which may hold therapeutic relevance for ICH recovery. Incorporating transwell and chip systems has enabled additional structural complexity to be replicated, and perfusion of media revealed morphological and functional differences in endothelial cells that are lacking in static models. Therefore, building a more complex, 3D, multisystem model is essential for recreating the aspects of ICH pathology that elude the capacity of *in vitro* models.

### 2.2 Transwells for recreating ICH pathology

**Fig. 3:**
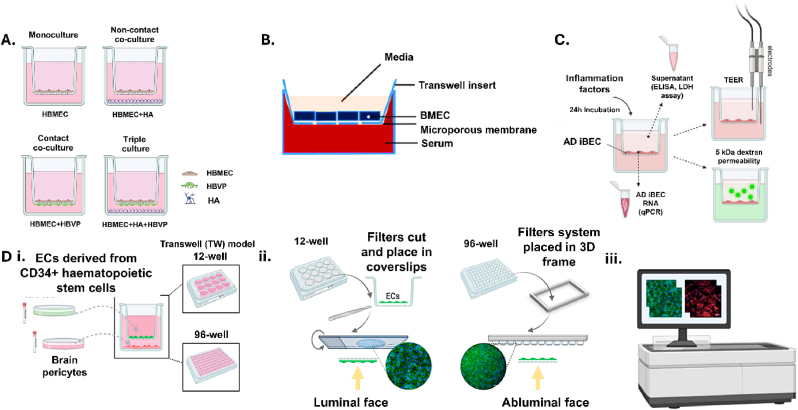
**Transwell models and the recreation of the neurovascular unit. A.** Schematic representation of the configurations for *in vitro* static human primary cell-based blood-brain barrier models. A monoculture model contains HBMEC seeded on the apical side of the well-insert porous membrane. A non-contact co-culture contains HBMEC seeded on the upper surface of the well-insert support and HA seeded at the bottom of the culture well. A contact co-culture model includes HBVP seeded on the lower surface of the well-insert support with HBMEC on the upper surface. For the triple culture model, HBMEC are seeded on the upper surface of the support with HBVP seeded on the lower surface and HA seeded on the bottom of the culture wells. Adapted from *Fattakhov* et al. (2022) [61]. HBMEC = human brain microvascular endothelial cells; HA = human astrocytes; HBVP = human brain vascular pericytes. **B.***in vitro* model of subarachnoid haemorrhage. The blood–brain barrier divides the system into two compartments (vascular side: upper compartment, brain parenchymal side: lower compartment). Medium was added to both compartments to prevent the cells from drying out. To simulate subarachnoid haemorrhage, the medium in the lower compartment was replaced by human serum to facilitate contact with the medium from above and the serum from below. Since the compartments are separated by a microporous membrane, the diffusion of small molecules (e.g., from the serum) is possible. Adapted from *Thal* et al. (2022) [65]. BMEC = human brain microvascular endothelial cells. **C.** Utilising the transwell model system to determine the effects of TNFα/IFNγ stimulation on the AD patient-derived BBB model, AD iBEC were cultured on Transwell inserts and TNFα/IFNγ were added to the top chamber of the Transwell insert. Following 24 h treatment, TEER measurement and 5 kDa dextran permeability assays were performed. Cell pellet and supernatant samples were collected for subsequent analysis with qPCR, ELISA, and LDH assays. Adapted from *Wasielewska* et al. (2024) [52]. BBB = blood-brain barrier; TNFα = tumour necrosis factor α; IFNγ = interferon gamma; ELISA = Enzyme-Linked Immunosorbent Assay; LDH = lactate dehydrogenase; TEER = transendothelial electrical resistance; AD = Alzheimer's Disease; iBEC = induced brain endothelial-like cell; qPCR = Quantitative PCR. **D. i.** Cells were thawed in petri dishes and let grown for 2 days. Then, cocultures were settled by seeding human ECs in the filters and brain pericytes in the bottom compartment either for 12-well plates or miniaturized 96 TW systems. **ii.** In the original 12 TW model, filters were cut and placed in coverslips, then returned to acquire the pictures over the luminal faces of the ECs. **iii.** Filters from the miniaturized systems were directly placed on a 3D frame developed and adapted to the microscope. Adapted from *Moya* et al. (2021) [51]. TW = transwell.

Models quickly developed to using transwells to create layers in static culture. The porous membrane of the transwell allows for nutrient and oxygen transfer between the internal and external well, and when seeded with endothelial cells, provides a basement framework for the cells to bind and interact (Table 1). There is use for mono- bi- and tri-co-culture models depending on the questions being addressed [38]. However, TEER measurements show transwell models are 1-2 orders of magnitude less resistant than *in vivo* measurements, and the lack of direct contact between cell types alters cellular phenotype questioning the validity of drug transport assays. Monocultures of endothelial cells have little relevance to the BBB as pericytes are required for endothelial cell maturation and tight barrier function [39] and the presence of astrocytes in the culture increases tight junction resistance [40]. To form the tri-culture model endothelial cells are seeded into the internal well, pericytes [41] or astrocytes [42,43] on the outer membrane and neurons in the bottom of the well (Fig. 3). This system allows for interactions between cells held on the porous membrane of the transwell and exposes the neuronal culture to signalling molecules potentially released into the media. This tri-culture model has the highest TEER values [44,45] and requires a cell ratio of 1:3 neurons to astrocytes [46]. Recently, the development of a four-cell transwell culture using primary human cells has surpassed the multi-cellular complexity of the tri-culture method by adding a neural cellular component and validated an increased physiological relevance with TEER and transcytosis assays [47,48]. There have been many published protocols utilising the commercially available human microvascular brain endothelial cells and immortalised cell lines, in addition to the differentiation of a human iPSC culture into multiple neural [46,49] and vascular cells types [50]. The transwell approach has been scaled to 96 well plates, in order to increase the reproducibility and validity of the model, and its application in high-throughput drug screening [51] (Fig. 3).

**Table 1:** Summary of the transwell models, uses of hydrogels/biomaterials, and microfluidic systems that are promising for ICH∗ research.

Model type	Model summary based on		Parameters assayed	Application for pre-clinical ICH modelling	Advantages	Limitations	Ref
	cell type	format					
Transwells	• Human brain primary astrocytes (ScienCell Research Laboratories) • Human brain primary pericytes (ScienCell Research Laboratories) • Human brain microvascular endothelial cells (ScienCell Research Laboratories) • Human neuronal cells (ScienCell Research Laboratories)	• Transwell four-cell culture • Astrocytes and pericytes in co-culture on the basolateral side • Endothelial cells on the apical side • Neurons cultured in wells	• TEER∗ • Confocal microscopy visualisation of cells • Drug transport	• BBB∗ behaviour in healthy controls • Drug transport • Cell death • BBB impairment • Axonal degeneration	• Multi-system complexity • All human cells	• High-risk culture strategy • 11-day culture	47
	• Human umbilical cord blood-derived endothelial cells • Bovine brain pericytes	• 96-well Transwell monoculture • Endothelial cells on the apical side • Pericytes in wells	• Molecular transport • Gene analysis of transporter and tight junction proteins	• Drug transport • BBB impairment • Metabolic changes	• Scalability for drug screening	• Mixed species culture • Limited cell type • No cell interactions	51
	• Human umbilical vein endothelial cells (HUVECs) • Human-induced pluripotent stem cells (hiPSCs) from controls and Alzheimer's disease patients	• Transwell monoculture	• TEER • Immunofluorescent characterisation • Cell viability • Metal accumulation and permeability • Pro-inflammation activity	• Patient-derived cells • Drug transport • Inflammatory profiling • Cell death	• Human cells • Patient-derived cells • Scalability for drug screening	• Lacks physiological complexity	52
	• Human-induced pluripotent stem cells (hiPSCs) from controls and childhood cerebral adrenoleukodystrophy patients	• Transwell monoculture	• TEER • Immunofluorescent characterisation • BBB diffusion permeability • Efflux transporter activity • Tight junction protein expression • Electron microscopy • Transcription analysis	• Patient-derived cells • Drug transport • Inflammatory profiling • Cell death • Genetic causes	• Human cells • Patient-derived cells • Scalability for drug screening	• Limited cell type	53
Transwells	• Human brain primary astrocytes (ScienCell Research Laboratories) • Human brain primary pericytes (ScienCell Research Laboratories) • Human brain microvascular endothelial cells (ACBRI 376)	• Transwell tri-culture • Endothelial cells on the apical side • Pericytes on the basolateral side • Astrocytes in the well	• TEER • BBB diffusion permeability • Hypoxia	• Ischaemia-reperfusion injury • BBB impairment • Metabolic changes • Cell death	• Human cells • Contact culture	• Limited cell types • Two-dimensional	61
	• Human brain microvascular endothelial cells (hCMVEC/D3) • Primary neutrophils from human blood	• Transwell monoculture	• Oxygen-glucose deprivation • Cell viability • immune cell interaction • BBB diffusion permeability	• Inflammation • Ischaemia-reperfusion injury • BBB impairment	• Human cells	• Limited cell type	63
	• Murine cerebellar endothelial cells (cerebEND)	• Transwell monoculture • Human blood serum	• Tight junction • Protein expression	• Tight junction response to patient serum • BBB impairment • Cell death	• Cardiovascular disease patient serum	• Mixed species culture • Limited cell type	65
Hydrogels and Biomaterials	• Rat astrocytes • Rat brain microvascular endothelial cells • Rat bone marrow derived endothelial progenitor cells • Human glioma cells (U118)	• 3-layer coaxial needle • Core of alginate/gelatin formed a hollow tube and coated in heparin-chitosan • Endothelial cells seeded on PLLA∗/gelatin scaffold and wrapped around a core of heparin-chitosan • Astrocytes seeded on a PLLA/gelatin scaffold and wrapped around endothelial cell layer • Core degraded after 7 days	• Scanning electron microscopy of cell morphology • Haematoxylin/eosin staining of cell morphology • Growth factor release • Tight junction protein expression	• BBB impairment • Vessel stability • Inflammation • Cell death	• Contact culture • Capacity for flow • 3-dimensional	• High -risk culture strategy • Mixed species culture culture	73
Hydrogels and Biomaterials	• Neutrophils isolated from human blood • Rat brain endothelial cells (RBE4)	• PDL∗ gel tube seeded with endothelial cells • Tube runs parallel to capillary array for generating a chemotactic gradient	• Oxygen-glucose deprivation • Cell viability • Tight junction protein expression • Neuroinflammatory markers	• Cell migration • Inflammation • Drug transport • Neuroinflammatory cell transcytosis • Cell death	• 3-dimensional	• Limited cell type • Single layer BBB • Restrictive gel barrier	74
	• Human brain microvascular endothelial cells (hBMVECs) • Human foetal glial cells (SVGP12)	• Y shaped GelMa microchannel • No barrier • Tissue layer contains SVGP12 cells in GelMa hBMVECs in 'vessel' formation in direct contact	• Cell viability • BBB diffusion permeability	• BBB impairment • Drug transport • Neuroinflammatory cell transcytosis • Hypertension • Cell death • Vascular stability	• Simple fabrication • Direct cell contact • Multiplex capabilities	• Limited cell type • Variables difficult to control for • Lack of brain complexity	75
	• Primary human brain microvascular endothelial cells (ACBRI 376) • Human immortalised monocytes (THP-1) • Human peripheral blood mononuclear cell isolated T cells	• Mimetas OrganoPlate® • hBMVECs lumenised top channel • ECM gel fills the central channel • empty bottom channel	• TEER • Tight junction protein expression • BBB diffusion permeability immune cell interaction	• Neuroinflammatory cell transcytosis • Drug transport • Inflammation • Vascular stability • Genetic causes of vascular weakness	• Commercially available • Simple culture	• Gravitational flow	79
	• Primary human brain microvascular endothelial cells (ACBRI 376) • Induced pluripotent stem cell-derived human astrocytes (Fujifilm-DI 01434) • Induced pluripotent stem cell-derived neural stem cells (Ax0018, Axol Bioscience)	• Mimetas OrganoPlate® • hBMVECs lumenised top channel • empty middle channel • astrocytes and neurons co-culture bottom channel	• Tight junction protein expression • Efflux transporter activity • Neuronal activity • TEER • Cell viability	• Oxygen-deprivation model • BBB impairment • Hypertension • Neuronal degradation • Inflammation • Cell death	• Commercially available • Neural cell types cultured in direct proximity	• Gravitational flow • Central channel separates endothelial and neural cell cultures	80
Hydrogels and Biomaterials	• Human umbilical vein endothelial cells (HUVECs, Lonza) • Human cerebral microvascular cells (hCMVEC/D3, Lonza) • Human brain astrocytes (ScienCell Research Laboratories) • Human brain pericytes (ScienCell Research Laboratories) • Human prostate cancer (PC3 and BT-474)	• Endothelial cells populate a 125 μm channel • Pericytes and astrocytes suspended in surrounding collagen type 1 gel • Cancer cells injected into 4-day cultures	• Cell viability • BBB diffusion permeability	• Angiogenesis in post-stroke patient cells • Hypertension • Neuroinflammatory cell transcytosis • Vascular stability • Genetic causes of vessel weakness • Inflammation • BBB impairment • Cell death • Metabolic changes	• 3-dimensional • Simple culture fabrication • Cells in direct contact to make the BBB structure	• No neuronal culture	82
	• Primary human brain microvascular endothelial cells • Human brain pericytes • Human brain astrocytes (ScienCell Research Laboratories)	• Astrocytes seeded into collagen gel • Pericytes and hBMVECs sequentially seeded onto cylindrical lumen	• BBB diffusion permeability • Pro-inflammation activity • Basement membrane changes	• BBB impairment • Drug transport • Hypertension • Angiogenesis in post-stroke patient cells • Inflammation • Cell death • Vascular stability	• Cells in direct contact to make the BBB structure	• No neuronal culture	83
	• iPSC-derived neurons • Human umbilical vein endothelial cells • Human umbilical cord myofibroblasts	• Smooth muscle cells, endothelial cells and astrocytes sequentially seeded into a PGA/PCL∗ porous scaffold • 60–80-day old neurons are seeded onto the scaffold surround in Matrigel • Circulating media for 3 weeks of culture	• Neuronal activity • Synaptic activity • BBB diffusion permeability • Tight junction protein expression	• Neuronal degradation • Drug transport • Cell death • Metabolic changes • BBB impairment	• Addition of smooth muscle cells • Cells in direct contact to make the BBB structure • Perfusion application for disease modelling	• High -risk culture strategy	86
	• Human endothelial cells (bEnd.3) • Human glioblastoma cells (U-87)	• 10 μm diameter porous tube printed using two-photon lithography • bEnd.3 seeded surrounding the tube structure	• Tight junction protein expression • BBB diffusion •Permeability TEER	• Hypertension • Spontaneous vessel rupture • Cell death • BBB impairment • Drug transport	• Biomimetic scale • Reproducible	• Synthetic tube structure and uniform pore size	91
Microfluidics	• Human brain endothelial cells (genetically modified in house) • Peripheral blood mononuclear cells (PBMCs)	• NeuroProbe AA12 chemotaxis chamber modified for flow • Endothelial cells seeded onto filter and cultured under flow • PBMCs injected into flow and transmigration into lower chamber analysed	• Immune cell transmigration	• Inflammatory profiling • Patient cells • BBB impairment • Metabolic changes • Genetic causes of vascular weakeness • Hypertension	• Patient-derived cells	• No neuronal culture • Large physiological distances • Synthetic filter barrier	97
	• Human primary astrocytes (Sanbio) • Human cerebral microvascular endothelial cells (hCMVEC/D3) • Peripheral blood mononuclear cells (PBMCs)	• Triple B slides designed in house • Endothelial cells seeded onto filter and cultured under flow • Astrocytes cultured on underside of the filter • PBMCs injected into flow and transmigration into lower chamber analysed	• TEER • BBB diffusion permeability • Tight junction protein expression • Immune cell transmigration	• Inflammatory profiling • Patient cells/blood • BBB impairment • Cell death • Transcytosis of immune cells • Hypertension	• Patient-derived cells • Adjustable chip design for 2D and 3D	• No neuronal culture • Synthetic filter barrier	100
	• Primary rat cortical neurons • Primary rat astrocytes • Human umbilical vein endothelial cells (HUVECs) • Human cerebral microvascular endothelial cells (hCMVEC/D3)	• Four channels seeded sequentially with endothelial cells, astrocytes, neurons and media	• Calcium imaging • BBB diffusion permeability • Neuronal development	• Neuronal degradation • Transcytosis of immune cells • Drug transport • Hypertension • Cell death • BBB impairment	• Perfusion	• Mixed species culture • Cells suspended in gel sections	101
	• Human brain microvascular endothelial cells (hBMVEC, ScienCell) • Human astrocytes (ScienCell) • Human brain vascular smooth muscle cells	• Lumenised endothelial tubes surrounded by either astrocytes (capillary model) or smooth muscle cells (venule model) on a perfused chip	•TEER •BBB diffusion permeability •Cellular metabolism	•Metabolic changes •Hypertension •BBB impairment •Drug transport •Cell death •Inflammation	•Perfusion •Variable control	•No neuronal culture •No pericytes	106
Microfluidics	• Human brain microvascular endothelial cells (hBMECs, ACBRI 376) • Human umbilical vein endothelial cells (HUVECs) • Primary human placental pericytes • Primary human astrocytes • Primary human lung fibroblasts	• Astrocytes and fibroblasts are seeded in fibrin gel into channels 2 and 4 separated by 650 μm • Endothelial cells and pericytes are seeded into channel 1 under flow • VEGF∗ is released by fibroblasts to promote cell migration and angiogenesis across the astrocyte gel	• Tight junction protein expression • BBB diffusion permeability	• BBB behaviour in healthy controls • Drug transport • Angiogenis repair • BBB impairment • Metabolic changes • Hypertension • Inflammation • Cell death	• Cell complexity • Variable control • Perfusion	• No neuronal culture	107
	• Human induced pluripotent stem cells • Primary human astrocytes • Primary human pericytes	• Organ-chip with two neighbouring channels, one seeded with endothelial cells and one a mixed culture of astrocytes and pericytes • Neuronal cells incorporated into brain side	• Neuronal activity • Tight junction protein expression	• Patient cells/blood • BBB impairment • Drug transport • Neuronal degradation • Cell death • Hypertension • Metabolic changes • Genetic causes of vascular weakness • Inflammation	• Perfusion • Direct cell interaction between astrocytes and endothelial cells • Self-assembling cell model • Potential for multiplexing • Primary and stem cell-derived cell populations commercially available	• Lacks cerebral complexity	108
	• Human primary astrocytes (ScienCell) • Transformed human microglia (HMC3) • Human induced pluripotent stem cell-derived neural progenitor cells • Human primary brain microvascular endothelial cells • Human brain vascular pericytes	• Three parallel channels on a chip comprising of endothelial cells and pericytes mixed culture in a 'blood' channel • Neural progenitor cells, astrocytes and microglia in a mixed culture in the central channel • Third channel, 'CSF∗' for media flow	• BBB diffusion permeability • Ischaemia response • Cell viability • Neuronal degeneration • Tight junction protein expression • Neuronal activity	• Inflammation • Neuronal degradation • BBB impairment • Hypertension • Oedema • Cell death	• Mixed cell culture in direct contact • Immune cells included	• Requires biomaterial optimisation • Gravitational flow	110
Microfluidics	• Human primary brain microvascular endothelial cells (hMVEC/D3) • Human brain astrocytes (ScienCell Research Laboratories)	• Hollow fibres seeded with mixed endothelial cell and astrocyte cultures	• Tight junction protein expression • BBB diffusion permeability • Pro-inflammation activity	• Inflammation • BBB impairment • Drug transport • Angiogenesis in post-stroke patient cells	• Perfusion • Mixed cells culture in direct contact • Easy model construction	• Lacks cerebral complexity • Lacks BBB morphology complexity	113
	• Primary human astrocytes • Primary human pericytes • Human hippocampal neural stem cells • Other cell lines to fabricate the other organ chips	• Multiplexed organ chips to complete a whole-body system (gut, liver, kidney, heart, lung, BBB, brain and skin). • Endothelial cells are seeded to line one channel and pericytes and astrocytes are co-cultured in the neighbouring channel to make the BBB chip • Astrocytes and neurons are co-cultured into one channel for the brain chip and the other channel is left for flow	• Tight junction protein expression • BBB diffusion permeability	• Drug transport • Drug toxicity • Genetic co-morbidities • Hypertension • BBB impairment • Neuronal degradation • Cell death • Gut-brain axis • Inflammation • Cardiac influence	• Whole body representation • Scalable	• Porous semi-permeable membrane separates channels • Very complicated culture protocol • Automation is lab-limited	114
	• Human primary brain microvascular endothelial cells (hMVEC/D3) • Human brain astrocytes (ScienCell Research Laboratories)	• Central basolateral compartment interconnected with outer apical compartments that are seeded with endothelial cells	• Tight junction protein expression • BBB diffusion permeability	• BBB impairment • Inflammation • Hypertension • Metabolic changes • Cell death	• Perfusion	• Lacks cerebral complexity • Lacks BBB morphology complexity	115
	• Human hippocampal neural stem cells • Cortical human brain microvascular endothelial cells (hBMVECs) • Primary human astrocytes • Primary human pericytes	• Endothelial cells are seeded to line one channel and pericytes and astrocytes are co-cultured in the neighbouring channel to make the BBB chip • Astrocytes and neurons are co-cultured into one channel for the brain chip and the other channel is left for flow	• Cell viability • BBB diffusion permeability • Cell metabolism and genetic profiling	• BBB impairment • Drug transport • Neuronal degradation • Metabolic changes • Inflammation • Cell death • Hypertension	• Coupled BBB and brain chip sections • Variable control • Potential for multiplexing	• Decoupled neurovascular unit	116

∗ICH: intracerebral haemorrhage, TEER: Trans-endothelial electrical resistance, BBB: blood-brain barrier.

∗PLLA: Poly(L-Lactic Acid).

∗PDL: poly-d-lysine, GelMa: gelatin methacryloyl.

∗PGA/PCL: poly(caprolactone-co-glycolide).

∗VEGF: vascular endothelial growth factor, CSF: cerebrospinal fluid.

Additionally, patient-derived stem cells [52,53] have been incorporated into these models. One such study highlights the phenotypic differences between cells acquired from different donors and found differences in terminal differentiation, expression markers and maturation [22]. These co-cultures are the most relevant, simplest *in vitro* model and therefore offers a good platform for preliminary study. They have been critical for the interrogation of immune involvement at the BBB in a static co-culture, as the open well allows for easy manipulation [54]. With respect to ICH modelling, the open transwell enables addition of blood to the endothelial layer and can be utilised to determine the toxicity of blood on different cell types however, lacking mature neuronal tissue limits this interpretation. Additionally, the physical distance between cell types at the bottom of the outer well does not have translational relevance to the physiological neurovascular unit.

The synthetic membrane prevents normal barrier architecture and cell-cell interactions, primarily due to the size constraints in manufacture and the lack of degradation essential for tissue remodelling. This has been addressed by attempting to design a biomimetic membrane using electrospinning to form polylactic-co-glycolic acid fibres [55] and bio-degradable membranes with gelatin [56,57], which highlights the need for biological materials for cellular scaffolds. Another iteration includes a device that is composed of a silicon nitride membrane (μSiM) to overcome the size limitations, forming a 100 μm barrier that is optically clear and permeable [58]. *Shima* et al. designed a membrane made from collagen ‘vitrigel’ formed through a dehydration step called vitrification, that was removeable from the insert frame to image cells [59]. Self-assembling, recombinant spider silk proteins have also been used to form a membrane that can be functionalised for optimal endothelial cell culture [60].

It is desirable to adapt these well-established models to ICH modelling, as there is a good understanding of the control state. However, the presence of the physical framework of the transwell membrane does not provide scope for a BBB rupture and the investigation of the cellular mechanisms behind the vascular weaknesses that lead to ICH. Some of these BBB models have been applied for stroke pathology study, exploring oxygen-glucose deprivation in ischaemia [[61], [62], [63], [64]] and blood insults of an ICH [65] (Fig. 3). Nevertheless, the lack of dynamics and structural complexity can only inform so far about the cellular response aspects of ICH pathology. The relevant research assessing immune cell interaction with the BBB, and drug transport across the membrane are appealing from a therapeutic perspective.

### 2.3 ICH models incorporating hydrogels and other biomaterials

To overcome the limitations of the transwell system, and allow for the additional complexity of the tri-dimensionality of the BBB [66], models that incorporate hydrogels and biomaterials provide an extracellular scaffold and interaction with essential extracellular matrix (ECM) molecules that direct cell behaviour [67] (Table 1). Biologically, the BBB ECM is comprised of perlecan, collagen IV and laminin and is linked by glycoproteins that can be replicated artificially in culture [68]. In this respect, appropriate materials should be mouldable, degradable and support cellular function. Most commonly, hydrogels are made of collagen I and IV, fibronectin, laminin and hyaluronic acid [69] to replicate the relevant ECM in the brain that provides both structural support for cells and biophysical cues for migration, proliferation and differentiation.

The incorporation of gels into human transwell models has advanced the structural complexity to mimic drug transport, which offers translational relevance when investigating potential therapeutics for ICH sufferers. Commonly used Matrigel, a basement membrane protein mix (majority collagen IV, laminin and perlecan) that is produced from mouse sarcoma cells, enables prolonged cell cultivation for up to two weeks when used to seed cells in a di-cell [70] and tri-cell cultures [59]. Despite Matrigel popularity, it is widely agreed across the field that due to variable composition and poor component definition, there is a need for well-characterised, tuneable alternatives [71]. Combinations of collagen microfibres and fibrin gel used to encapsulate endothelial cells, astrocytes and pericytes have allowed for spontaneous self-assembly into microvasculature structures. Such models in 96 well plates are easily scalable for model development and interrogation of injury mechanism to the BBB [72].

**Fig. 4:**
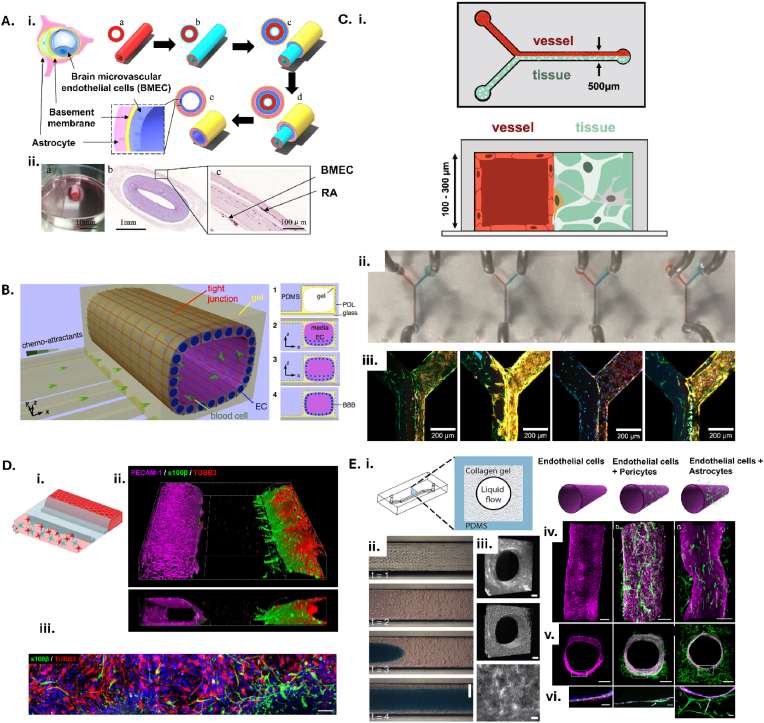
**Utilising hydrogels to add 3D complexity to models of the blood-brain barrier.A.** The biofabrication process of the *in vitro* BBB model. (a) Step 1, gelatin and sodium alginate were extruded by coaxial needle, which contained growth factors. (b) Step 2, heparin-chitosan electrostatic self-assembled coating, this core–shell scaffold structure played a role in controlled release of the growth factors. (c) Wrapped endothelial cell layer. (d) Wrapped astrocyte layer, (e) after culture and induction, the internal scaffold gradually degraded and tight junctions were formed between endothelial cells. **ii.** (a) Overall optical photograph, (b) Haematoxylin and eosin staining of the cross-section, (c) Local magnification. Adapted from *Liu* et al. (2020) [73]. BMEC = human brain microvascular endothelial cells; RA = rat astrocytes. **B.** A 3D BBB model consists of EC arranged in a cylindrical monolayer, forming a tight seal and separating a “blood” compartment from an array of capillaries presenting chemotactic gradients. The 3D BBB model is constructed by (**1**) coating an adhesive molecule, PDL and a gel on PDMS microstructures and a glass substrate, (**2**) plating brain EC first on the top and (**3**) later on the bottom surfaces and (**4**) culturing to form tight monolayers. Adapted from *Cho* et al. (2015) [74]. PDL = Poly-D-lysine; PDMS = polydimethylsiloxane; EC = endothelial cells; BBB = blood-brain barrier. **C. i.** The BTI Chip. A blood vessel endothelium contacts a cell-embedded GelMA-PEG hydrogel*.***ii**. Parallel laminar flow profiles can be established simultaneously in multiple Y channels within the same PDMS housing. **iii.** Immunofluorescence micrographs of HDFn-embedded gels and hBMVEC endothelium demonstrate successful fabrication of an array of blood-tissue interfaces. Staining for VE-Cadherin (red) distinguishes hBMVEC from HDFn (phalloidin-stained actin, green; DAPI-stained nuclei, blue). Adapted from *Young* et al. (2023) [75]. BTI = blood-tissue interface; PDMS = polydimethyl-siloxane; GelMA-PEG = gelatin methacryloyl polyethylene glycol; HDFn = human dermal fibroblasts, neonatal; hBMVEC = human brain microvascular endothelial cells; DAPI = 4′,6-diamidino-2-phenylindole. **D. i.** 3D artist impression of the NVU on-a-chip model in the OrganoPlate 3-lane culture platform. **ii.** 3D reconstruction of the human NVU model showing a vessel of brain endothelial cells (PECAM-1, magenta) grown against an extracellular matrix gel, in co-culture with networks of astrocytes (s100β, green) and neurons (TUBB3, red). **iii.** Astrocytes (s100β, green) and neurons (TUBB3, red) are present in the bottom lane of the chips and form networks. All images were acquired from 14-day old cultures. Scale bars are 50 μm. Adapted from *Wevers* et al. (2021) [80]. **E. i.** A cross-section through the chip showing the PDMS channel containing the collagen gel made with viscous fingering and a central lumen. **ii.** Time-lapse images of the fingering method (bar, 500 μm). **iii.** Second harmonic generation image of the collagen distribution in the 3D BBB chip, an intensity generated voxel illustration of the lumen based on this information (bar, 100 μm), and a high magnification of the second harmonic generation image showing of collagen microstructure in the cylindrical gel within the 3D BBB chip (bar, 50 μm). **iv.** fluorescence confocal micrographs of the engineered brain microvessel viewed from the top, and cross-section at either low (**v.**) or high (**vi.**) magnification. Green indicates F-actin staining, blue represents Hoechst-stained nuclei, and magenta corresponds to VE-Cadherin staining. Adapted from *Herland* et al. (2016) [83].

Use of hydrogels also allows for the tuneable fabrication of 3D scaffolds beyond what is possible with transwells. *Liu* et al. utilised co-axial extrusion of sodium alginate and gelatin to produce a hollow tube-like structure that was immersed in alternating chitosan and heparin solutions and covered with an electrospun membrane seeded with endothelial cells and endothelial progenitor cells [73] (Fig. 4). After 7 days of culturing there was the induction of cell differentiation and the formation of tight junctions. A different tube-shaped approach by *Cho* et al. included the use of poly D-lysine solution and collagen type I incubation of the polydimethylsiloxane (PDMS) created tube to add an ECM support for endothelial cells [74]. The authors found the BBB model to replicate functional features in 3D, but such models would only be useful to investigate the pathology of ICH on the vascular cells. Ultimately, the design of the 3D tube structure fails to incorporate an adjacent space to add neural cells. *Young* et al. utilised stop flow lithography to generate two linear compartments in a Y-shaped device that could be multiplexed together in parallel to demonstrate reproducibility with characterised vessel function [75]. However, the latter did not characterise the model in the context of any disease (Fig. 4).

Hydrogels have allowed for the formation of layered models without the need for porous membranes to separate cell types as in the transwell system. Such innovative example includes the MIMETAS OrganoPlate® that incorporates phase guides to separate neural-cell loaded hydrogel from the fluid phase perfusing media. Polymerisation of the collagen hydrogel provided a surface on which to seed a layer of endothelial cells, recreating the layers of the BBB. Importantly, the lack of membrane in this model allows for shear stress of perfusing media to influence the cells held in the collagen hydrogel [[76], [77], [78]]. The MIMETAS OrganoPlate® has also been used to investigate T cell extravasation under pro-inflammatory conditions to determine migration from tissue necrosis factor (TNF)-α in the inflamed vessel component to CXC motif chemokine ligand (CXCL) 12 in the neighbouring space [79]. Additionally, ischaemic stroke was mimicked using oxygen/glucose deprivation and halting perfusion of the plate [80] (Fig. 4). A combination of such disease conditions caused the leakage of dextran through the barrier, reduced mitochondrial membrane potential and lowered adenosine triphosphate (ATP). This commercially available plate also allows for bi-directional gravitational flow when mounted to a plate rocker and offers additional sample wells compared to competitive products such as the AKITA Plate [81]. Although this induced flow may offer advantages for immune cell interaction modelling, a future upgraded model that tackles the physiological relevance to vascular shear stress would be beneficial.

Introducing hydrogels with adaptable stiffness allows tighter control over the ECM-like properties. Such strategy was exploited by *Tourovskaia* et al. to investigate spontaneous angiogenesis into a collagen hydrogel encasing neural cells in suspension [82]. This resulted in sprouting of lumenised vessels from an engineered central vessel seeded with human umbilical vein endothelial cells (HUVECs). The surrounding collagen gel also provided a space to investigate extravasation, in this case of cancer cells, but would also offer a platform for neuroinflammatory studies. *Herland* et al. generated a similar capillary tube in a collagen I gel before introducing endothelial cells and pericytes to coat the inner surface [83] (Fig. 4). The authors tested for vessel permeability with different cell combinations and observed leakage of dextran into the surrounding hydrogel. Notably, a different response to pro-inflammatory stimulus with TNF-α when compared to the static transwell culture was recorded, which was modulated by the inclusion of astrocytes/pericytes, highlighting the importance of the co-culture method for immune studies. Both models could be directly applicable to ICH studies, as the gel space surrounding the vessels could be used to observe blood cell breakdown, immune cell migration, and neuronal responses to vascular leaking.

Fibrin gels have been shown to allow for vasculogenesis when seeded with endothelial cells, astrocytes and pericytes, and vessels show maturity by depositing basement membrane proteins laminin and collagen IV and increasing expression of tight junction proteins [84]. Importantly, when fibrin gels were compared with different gels mixed with Matrigel or hyaluronic acid, it was found that fibrin-Matrigel mixed gel promotes more neuronal differentiation. The development of 3D networks of neurons was due to the presence of laminin, a prominent component of Matrigel [85]. The inclusion of smooth muscle cells with the endothelial cell culture in a biomaterial-based construct might seem attractive but adds an extra layer of complexity. This was the case when a biomaterial mesh tube was seeded internally with vascular cells (endothelial cells and smooth muscle cells) and externally with neurons and astrocytes [86]. The technical demand of generating such a model makes it difficult to scale up or translate to other labs. However, more complex models of the neurovascular unit are needed for accurate understanding of drug transport, diffusion and the interaction with nano-delivery devices. In addition, an increased control over the scaffold and structure, vessel formation, cell density, tube size, with a high level of reproducibility is required. Hollow fibre membranes of poly-(ɛ-caprolactone) were shown to be a highly permissive scaffold that allows the curvature of the vessels to be mimicked *in vitro* [87]*.* The structures exhibited good electrical conductivity and biocompatibility with cells of the BBB. However, when fabricated with graphene nanoplatelets, this negatively impacted the growth and viability of the endothelial cells. Graphene has been investigated in recent years for interesting biomedical applications [88], including applications in stroke [89,90]. Similarly, *Marino* et al. used two-photon lithography to generate a porous microtube with a biologically relevant scale of a brain capillary, with 2 μm pores, and a vessel diameter of 10 μm^91^. When seeded with mixed species mouse endothelial cells and human neural cells they observed stable tight junctions forming an impermeable lumen within the microtube. Such designs are suitable for multi-plexing [92], aiding the validation of results and the reproducibility of engineering a scaffold structure with is attractive to standardising the size of the capillaries that are modelled for a neurovascular unit, and can be adapted to modulate parameters such as pore size and density.

### 2.4 Microfluidics advances for ICH models

Shear stress is very important for accurately modelling cell morphology and behaviour and microfluidic systems play an essential role to provide added complexity and translational relevance to BBB models [93] (Table 1). Endothelial cells express markers and morphology that are more similar to *in vivo* conditions of the capillary wall when cultured under laminar flow. Flow, in the context of ICH injury where vessel rupture causes flow disruption, is a key factor of the disease state, responsible for intracranial pressure increase, rebleeding and clearance of the heamatoma [94]. The mechanisms that lead to the microvascular vulnerability and spontaneous rupture of blood vessels in the human brain are not well defined. These microfluidic systems would provide a platform to investigate the effects of hypertension in a BBB model, the leading cause of adult ICH. Some systems, such as the MIMETAS OrganoPlate® that operate using gravitational force to flow media across the chip are beneficial to make the system user-friendly and ease of manipulation for addition of drugs. However, in the absence of additional pressure induced by a pump system, there is no shear stress equivalent to physiological levels [76,95]. The MIVO® platform from React4Life incorporates fluidic flow into the traditional transwell system and offers additional physiological relevance recently employed for investigating drug delivery into glioma cells [96]. The incorporation of patient-derived cells into these complex models under flow would allow for personalised medicine, with the spontaneous differentiation of neural immune cells (microglia) and the epigenetic programming of endothelial cells that might help elucidate causes for vascular weakness and rupture in ICH patients.

Many fluidic chips that have been developed to build upon the basic structure of a channel for vascular-type cells and a channel for neural-type cells to be fed by two different media separated by a porous membrane. Building on the transwell model of using a physical porous membrane to divide cells, *Man* et al. designed a dual-layer perfusion chamber to investigate cellular transmigration under flow [97]. Flow of media allows for leukocyte rolling and attachment and transcytosis to be observed under shear stress [98], and could provide valuable insights into the immediate neutrophil response to ICH injury. The role of T cells in long term recovery after a stroke is very interesting for therapeutic targeting and most animal models of late disease are unsuitable for mimicking the human condition. *Lauranzano* et al. showed that T effector/memory cells were able to migrate across a co-culture of astrocytes and endothelial cells and the filter of a transwell, upregulated by tumour necrosis factor (TNF)-α [54]. Such models have also been used to investigate receptor transcytosis, along with their application for neurotherapeutic bioavailability studies [99]. *Meena* et al. designed a similar chip that could be multiplexed, so that parallel experiments could be run on the same pump system and determined that under flow conditions leukocytes exhibited ∼25 % less migration across the cell membrane [100].

**Fig. 5:**
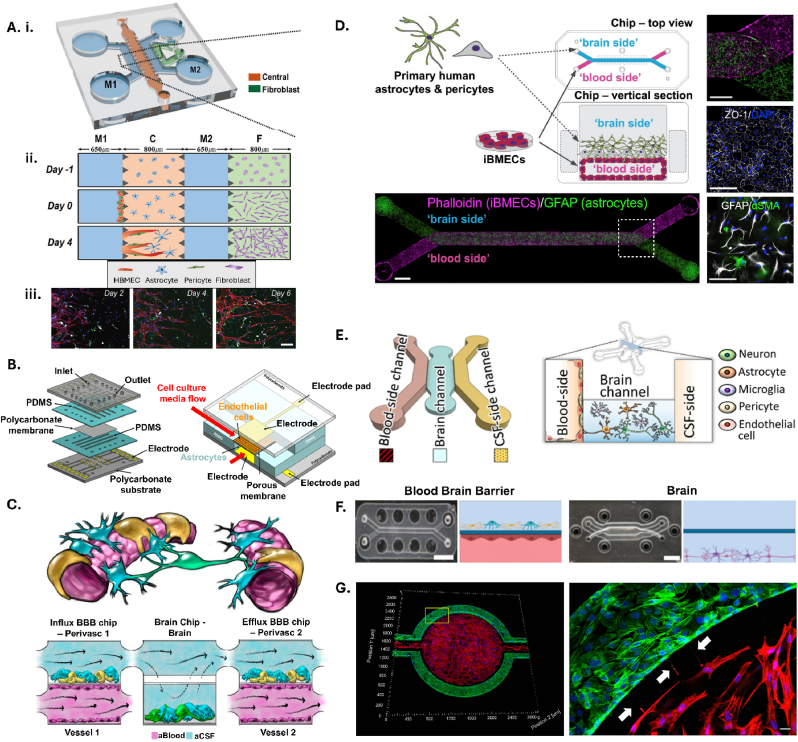
Approaches utilising microfluidics to add flow and enable shear stress. **A. i.** Schematic view of a microfluidic device having three types of microfluidic channels. **ii.** Section view of microfluidic device describing sequential loading and culture progress of BBB. Channel C (red) is the region where the final BBB microenvironment is constructed. Channel F (green) possesses 3D fibroblasts, which acts as a source of angiogenic factors. Channel M1 and M2 (blue) are media channels. **iii.** Day by day confocal imaging of channel C where HBMEC (anti-CD31, red) sprouts from the left end to right end. Astrocytes (anti-GFAP, white) protrude to generate end-feet and pericytes (anti-αSMA, green) wrap around EC as the day goes by. Nuclei were stained with Hoechst 33342 (blue); scale bar = 100 μm. Adapted from *Lee* et al. (2020) [107]. BBB = blood-brain barrier; HBMEC = human brain microvascular endothelial cells. **B.** The developed multi-channel multi-layer BBB chip with integrated electrical impedance sensor array for TEER analysis. Adapted from *Jeong* et al. (2017) [102]. BBB = blood-brain barrier; TEER = transendothelial electrical resistance; PDMS = polydimethylsiloxane. **C.** Simplified anatomical illustration of the neurovascular unit, highlighting positions and cell–cell interactions between vascular endothelial cells (pink) that line brain blood microvessels (left, right) and surrounding perivascular pericytes (yellow) and astrocytes (blue) that form the BBB, as well as neighbouring neurons (green) in the brain parenchyma and a schematic of the experimental setup of NVU system. Adapted from *Maoz* et al. (2018) [116]. BBB = blood-brain barrier; aBlood = artificial blood; aCSF = artificial cerebrospinal fluid. **D.** Schematic of BBB-Chip seeding paradigm, with iBMECs seeded on the blood side and primary human astrocytes and pericytes seeded on the brain side. Immunocytochemistry 5 days post-seeding shows the blood side is populated with iBMECs that form a monolayer and express phalloidin (magenta). The brain side is seeded with primary human astrocytes that express GFAP (green) and pericytes. Scale bar, 1 mm. High magnification images show expression of membrane ZO1, GFAP and α-SMA. Adapted from *Vatine* et al. (2019) [108]. iBMECs = induced pluripotent stem cell derived brain microvascular endothelial-like cells; ZO1 = zona occludens 1; GFAP = glial fibrillary acidic protein; α-SMA = α smooth muscle actin. **E.** The chip design and spatial distribution of the neurovascular unit constituent cells in the chip. Adapted from *Lyu* et al. (2021) [110]. CSF = cerebrospinal fluid **F.** Human organs for the Human-Body-on-Chip (HuBoC), with a representative Organ Chip photograph and sectional schematic. Adapted from *Novak* et al. (2020) [114]. **G.** Coculture of hCMEC/D3 and primary human astrocytes in μHuB. hCMEC/D3 monolayers (green) were cultured in the vascular (apical) compartments with primary human astrocytes (red) in the tissue (basolateral) compartment (nuclei, blue). Adapted from *Brown* et al. (2019) [115]. hCMEC/D3 = human brain microvascular endothelial cells.

Additional complexity allows for different cell types/media/section repeats which allow for built-in controls. *Adriani* et al. utilised 4 separate channels for seeding various cell types, including neurons, endothelial cells and astrocytes in hydrogel with tailored collagen gel concentrations for the different cell properties [101]. *Jeong* et al. built a chip with a co-culture of primary brain microvascular endothelial cells and astrocytes on four rows and columns of micro channels which resulted in sixteen intersecting regions where cells interact with an integrated electrical sensor for real-time TEER analysis [102]. The authors included a Matrigel basement membrane which yielded significantly tighter junctions between wells and higher TEER values (Fig. 5). A chip with built in sensors to measure TEER in real-time allowed the researchers to record responses to histamine in mono- and co-culture models and would provide an excellent platform to visualise cell responses to ICH risk factors such as low cholesterol and hypertension and understand the trigger point that leads to vessel rupture in ICH under flow.

Physiological flow involves the consideration of a multitude of different parameters beyond the size of the vessels, which are critical especially in disease conditions. Hypertension is the leading cause of ICH clinically and can occur in response to atherosclerosis and vessel constriction, for which many patients receive medication, that can alter the flow of red blood cells (RBCs) in circulation [103]. *Bouhrira* et al. designed a model for pulsatile flow that mimics systolic pressure changes along the waveform [104]. They characterised the effect of these dynamics on cell tight junctions and barrier permeability and observed that disturbed flow and inconsistent shear stress caused differences in cell function. This study not only highlights the importance of reproducibility in microfluidic studies, but of the disease parameters that contribute to the vessel weakness in ICH that are not easily replicated in the current *in vitro* models. It is critical to consider that the morphology of the cerebral vessels is not linear with further implications in flow pattern perturbations and subsequent vulnerability to rupture. To generate disturbed blood flow *in vivo*, the ICH model would need to be induced in a co-morbidity model, which is rarely applied [105]. *Cucullo* et al. fabricated a dynamic model of capillary (endothelial cells and astrocytes) and venules (endothelial cells and smooth muscle cells) to generate a perfusable system under pulsatile flow that models the BBB and the distal venule sections of the cerebrovasculature [106]. The latter provides an insight into the effects that different vessel physiology has on the ICH pathology, immune response and bleed severity, but it will have to be coupled with cerebral tissue to determine a translational effect.

Specific to central nervous system (CNS) angiogenesis, and the simultaneous formation of the BBB with angiogenic sprouting *in vivo*, *Lee* et al. designed a microfluidic chip to incorporate three types of human vascular cells and astrocytes with sequential seeding of fibroblasts to provide angiogenic factors and initiate sprouting [107] (Fig. 5). A fibrin hydrogel was used to mimic the ECM derived from bovine fibrinogen. With an optimised chip, vasculogenesis across a channel between fibroblasts and vascular cell chambers allow for the possibility of including neuronal cells in this space. Alternatively, there is reasonable optical access to investigate the leaking of these cells, and possibly immune cell interaction with the astrocytes of the neurovascular unit.

Organ-Chip from Emulate such as Chip-R1™ utilises two parallel microchannels that are separated by a porous membrane, similar to the transwell system, but allows for dynamic culture. *Vatine* et al. utilised the Organ-Chip to characterise the neurovascular unit interaction, observing astrocytes protruding through the pores to contact the vascular cells and selective transport of transferrin [108] (Fig. 5). Interestingly, to test functionality, the authors perfused with cytokines characteristic of vascular inflammation and observed a reduction in the pericyte and astrocyte contact with endothelial cells, offering an insight into a mechanism of vessel rupture that may occur in ICH patients. Notably, when seeding the model with patient cells carrying the genetic irregularity in the *HUNTINGTIN* gene they observed considerably more molecular permeability. Key to this study, the system was perfused with whole human blood to demonstrate blood-induced cytotoxicity in the absence of a functional BBB and demonstrated a valuable and pivotal model for direct application in ICH study. Other groups have utilised the Emulate chip coated in laminin, collagen IV and fibronectin to investigate barrier permeability [109]. Such a model, that recreates the microphysiological ECM, shear stress, cell complexity and toxicity caused by blood, offers a suitable and accessible fundamental platform for ICH investigation, and has proven viable with the incorporation of patient-derived cells. To support the relevance of these models in pre-clinical stroke research, *Lyu* et al. employed a microfluidic chip system to replicate the stroke damage in a model of ischaemia by recreating the penumbral region that is most highly targeted for therapeutics [110]. A central channel contained neural cells, with a ‘blood-side’ and a ‘cerebrospinal fluid (CSF)-side each flanking the brain embedding hydrogel (Fig. 5). Ischaemia was induced by depleting oxygen, glucose and serum for 24 h and through gene analysis, an upregulation of inflammatory cytokines and integrin expression, and a downregulation of ECM proteins was noted. This model platform was further used to investigate stem cell therapy as a restorative treatment following hypoxia, by injection into the ‘blood-side’ of the chip, recreating the cell attraction and adhesion to the vessel wall.

Comparing a culture condition in a chip to a transwell system as a static control introduces too many variables for a viable control. In some cases, complex intricate systems have too many variables and render it often unfavourable for large-scale screens. Manipulation of the cultures within the chip can cause bubbles or contamination. Sizes of the chambers and membranes separating sections are often not representative of the physiological environment [111]. Some studies attempt to address these limitations such as μBBB model that boasts a comparatively thin membrane of 10 μm and has been used with mouse cells [112] to better capture physiological levels of TEER and cell layer permeability. Additionally, a chip designed by *Moya* et al. aims to overcome some of the restrictions to experimental design and availability of controls by including the attunable sections all in one chip to provide control conditions in the same system [113].

Linking chips together can increase the complexity of the system and allow for multiple body systems to be included in the same experimental setup. *Novak* et al. developed a robotic platform to link intestine, liver, kidney, heart, lung skin, BBB and brain organ chips together for 3 weeks, which allowed for toxicity studies, off-target effects and hepatotoxicity of therapeutics to be investigated [114] (Fig. 5). The three‐dimensional human BBB microfluidic model (μHUB) model [115] utilises a commercially available, modular chip that showed physiological shear stress, selective permeability and structure allowing for spatial and temporal permeability changes to be determined, which could allow for study of the effect of blood toxicity over time and the downstream effect of the damage on subsequent vessels. *Maoz* et al. linked three separate chips to model BBB influx, parenchymal tissue and BBB efflux, using different media for the endothelial cell layer and the brain microtissue layer [116]. This dynamic model would be interesting to investigate the passing of RBCs through the different tissue types and give insight into the clearing of RBCs from the parenchyma along with the role of CSF flow compared to microglia.

## 3 3D models of vascular and cerebral tissue

### 3.1 Vascular spheroids and organoids for ICH modelling

Although different neural cell types have been seeded into these models most optimisation has focussed on flow and replicating the microphysiology of the neurovascular unit, rather than the maturity of the brain tissue. Spheroids: 3D self-assembled structures from non-adherent cell types, generated from vascular (pericytes, endothelial cells) and neural cells, and organoids: cultures of different cell types in 3D to form a microtissue (Table 2), have been used to investigate both cerebral and vascular tissue complexity [117] with relevance for ICH.

**Table 2:** Summary of the vascular spheroids/organoids, vascularised brain organoids, and perfusion of vascularised organoids models promising for ICH∗.

Model type	Model summary based on		Parameters assayed	Application for pre-clinical ICH modelling	Advantages	Limitations	Ref
	cell type	format					
Vascular spheroids/organoids	• ETV2∗-inducible endothelial cells from human induced pluripotent stem cells • Pericytes from human induced pluripotent stem cells • Neural progenitor cells from human induced pluripotent stem cells • Astrocytes from human induced pluripotent stem cells	• Cell types are mixed and embedded in ECM∗ gel for two weeks for spontaneous BBB∗ formation	• Cell morphology and interactions • Tight junction protein expression	• BBB Impairment • Vessel stability • Neuronal degradation • Genetic causes of vascular weakeness	• Scalable • Reproducible • Self-assembling cell model	• No neuronal culture • Variables difficult to control for	121
	• Human brain astrocytes (ScienCell Research Laboratories) • Human brain microvascular pericytes (ScienCell Research Laboratories) • Human cerebral microvascular endothelial cells (hCMEC/D3)	• Cell types mixed and seeded into micropatterned hydrogel Gri3D plates and form organoids	• Transcytosis • BBB permeability	• BBB impairment • Metabolic changes • Cell death	• Reproducible • Highly scalable • Self-assembling cell model	• No neuronal culture • Heterogenous organoid formation	122
	• Immortalised human umbilical vein endothelial cells • Primary human umbilical vein endothelial cells • Normal human lung fibroblasts	• Fibroblasts and endothelial cells mixed and seeded in fibrin gel into inlet channel • Channel runs alongside rhomboidal chamber for spontaneous tube formation	• Vessel morphology • AVM∗ formation • BBB permeability • Tight junction protein expression	• AVM modelling/therapy • Genetic causes of vascular weakness • BBB impairment • Inflammation • Cell death • Spontaneous rupture • Microbleeds	• Scalable • Adaptable disease phenotype	• No neuronal culture •Lacks cellular complexity of BBB	123
	• Human induced pluripotent stem cells	• Stepwise differentiation of stem cells into vessel networks and organoids	• Vessel morphology	• BBB impairment • Angiogenesis in post-stroke patient cells • Cell death • Genetic causes of vascular instability • Metabolic changes	• Scalable • Self-assembling cell model • Potential for patient-derived cells	• No neuronal culture	124
Vascularised brain organoids	• Human induced pluripotent stem cells	• Stepwise differentiation of stem cells to cerebral organoids. • VEGF∗ added during embryoid body formation for parallel endothelial cell differentiation	• Vascular cell differentiation	• BBB impairment • Angiogenesis in recovery • Inflammation • Neuronal degradation • Genetic causes of vascular instability • Cell death • Microbleeds	• Self-assembling cell model • Scalable • Long term culture for increased neural complexity	• Variable mesodermal differentiation • Long term culture • Lacks cellular complexity of BBB	127
	• Astrocytes derived from human induced pluripotent stem cells • Human brain microvascular endothelial cells (hBMVECs) • Human brain vascular pericytes (ScienCell) • Microglia (C1110)	• Neural progenitors, astrocytes and microglial are co-cultured for 2 weeks • Pericytes and hBMVECs were added in suspension and spheroids were cultured for 2 weeks	• BBB cell formation • Tight junction protein expression • BBB permeability	• Drug transport • Inflammation • Neuronal degradation • Cell death • Metabolic changes • Genetic causes of vascular instability • Microbleeds	• Quicker culture than cerebral organoids	• Lacking maturity • Basic microstructure of BBB	128
	• H9 human embryonic stem cells	• Stepwise differentiation of stem cells to cerebral organoids. • Stepwise differentiation of stem cells into vessel networks and organoids • Cerebral and vascular organoids are fused 1:2	• Vessel morphology • Tight junction protein expression • BBB permeability	• Inflammation • Drug transport • BBB impairment • Angiogenesis post-stroke • Cell death • Genetic causes of vascular weakness • Microbleeds	• Spontaneous cell differentiation and maturation • Reproducible • Multisystem complexity	• High -risk culture strategy	130
	• Human induced pluripotent stem cells	• Stepwise differentiation of stem cells into vessel networks and cerebral organoids. • Day 15 vascular organoids are dissociated and co-cultured with cerebral organoids for 2 days before embedding	• Vessel morphology • BBB permeability	• Drug transport • BBB impairment • Angiogenesis post-stroke • Genetic causes of vascular instability • Cell death • Inflammation • Neuronal degradation • Microbleeds	• Spontaneous cell differentiation and maturation • Reproducible • Multisystem complexity	• Lacking astrocytes • High -risk culture strategy	134
	• H9 human embryonic stem cells • Human induced pluripotent stem cells • Cerebral cavernous malformation patient cells	• Stepwise differentiation of stem cells to cerebral organoids. • Initiation of astrocyte differentiation at day 60 • Cerebral and vascular organoids are fused 1:1	• TEER∗ • Tight junction protein expression • BBB permeability	• Drug transport • Inflammation • BBB impairment • Angiogenesis post-stroke • Genetic causes of vascular instability • Cell death • Neuronal degradation • Spontaneous rupture • Microbleeds	• Spontaneous cell differentiation and maturation • Reproducible • Multisystem & microtissue complexity	• High -risk culture strategy	133
Perfusion of vascularised organoids	• Human embryonic stem cells	• Stepwise differentiation of stem cells to cerebral organoids with ETV2 induction for simultaneous endothelial cell differentiation	• BBB permeability • TEER • Perfusion • Cell viability • Tight junction protein expression • Implantation into mouse	• Inflammation • Drug transport • BBB impairment • Neuronal degradation • Hypertension • Co-morbidities *in vivo* • Cell death • Oedema • Spontaneous rupture	• Self-assembling cell model • Spontaneous cell differentiation and maturation • Reproducible • Multisystem complexity • Microtissue complexity • Perfusable	• High -risk culture strategy • Long term culture • Scalability for drug screening	136
	• Human induced pluripotent stem cells	• Stepwise differentiation of stem cells to cerebral organoids. • Stepwise differentiation of stem cells into vascular cells • Co-culture on chip at day 7	• Perfusion • Vessel morphology	• BBB impairment • Inflammation • Genetic causes of Vascular instability • Neuronal degradation • Cell death • Hypertension • Spontaneous rupture • Metabolic changes • Microbleeds • Targeted lesions • Recovery • Drug transport	• Scalability for drug screening • Self-assembling cell model • Spontaneous cell differentiation and maturation • Multisystem complexity • Perfusable • Microtissue complexity	• Reproducibility - multiple chips developing individually • Long term culture	137
	• Human induced pluripotent stem cells	• Stepwise differentiation of stem cells to cerebral organoids • Day 2 spheroids implanted into soft, permeable microfluidic grid	• Gene expression • Cell viability • Perfusion	• BBB impairment • Inflammation • Genetic causes of Vascular instability • Neuronal degradation • Cell death • Hypertension • Spontaneous rupture • Metabolic changes • Microbleeds • Targeted lesions • Drug transport	• Spontaneous cell differentiation and maturation • Perfusable • Scalability for drug screening	• Lacks cellular complexity of BBB	138

∗ICH: intracerebral haemorrhage, ETV2: Ets variant 2, ECM: extracellular matrix, BBB: blood-brain barrier, AVM: arterio-venous malformation.

∗VEGF: vascular endothelial growth factor, TEER: trans-endothelial electrical resistance.

**Fig. 6:**
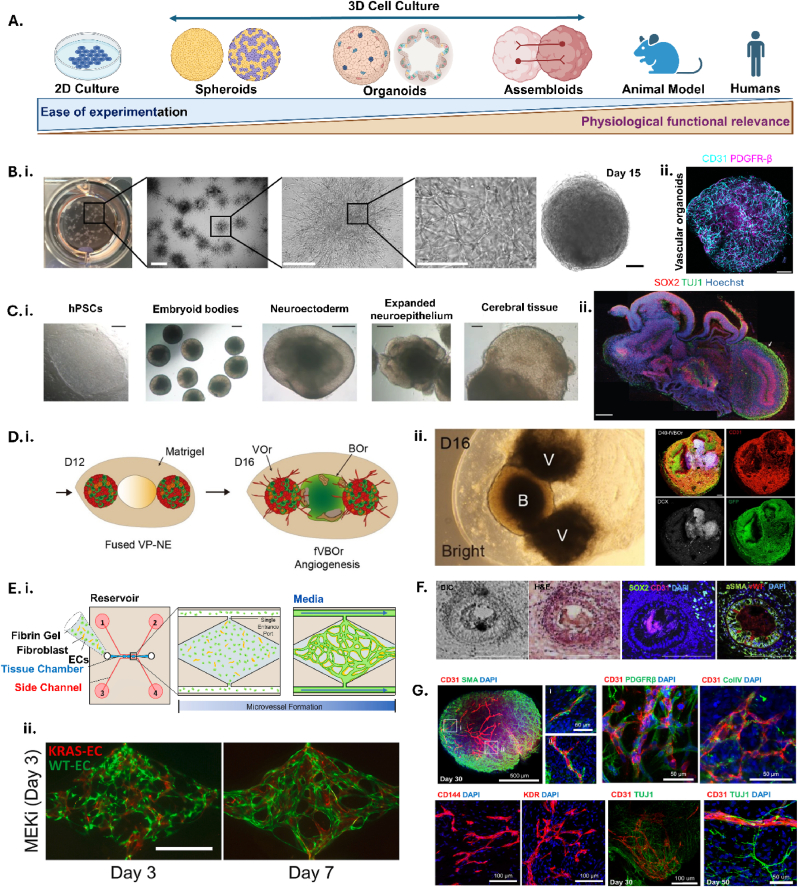
**Spheroid/organoid models of neurovasculature**. **A**. Increasing microtissue complexity increases physiological relevance to the human condition however, become more experimentally complex. Created in BioRender. Lomora, M. (2025) https://BioRender.com/1d60m3q. Adapted from *Lee* et al. (2023) [117]. **B. i.** Culture protocol for developing a blood vessel organoid **ii.** A mature blood vessel organoid at day 15 is stained for endothelial cells (CD31, cyan) and pericytes (PDGFR-β, magenta). 50um scale. Adapted from *Wimmer* et al. (2019) [124]. **C. i.** Culture protocol for developing a cerebral organoid. **ii.** Sectioning and immunohistochemistry revealed complex morphology with heterogeneous regions containing neural progenitors (Sox2, red) and neurons (Tuj1, green) (arrow). Adapted from *Lancaster* et al. (2013) [125]. **D. i.** Schematic of the method for generating fVBOrs. **ii.** fVBOrs at different developmental stages. V, VOr; B, BOr. Immunostaining of CD31 and DCX for labelling vessels and neurons, respectively, in day 40 fVBOrs. Scale bar, 200 μm. Adapted from *Sun* et al. (2022) [130]. EBs = embryonic bodies; NE = neuroepithelium; VP = vascular progenitor; VO = vessel organoid; BOr = brain organoid; fVBOrs = fused vascular and brain organoid. **E. i.** Schematic representation of device developed for modelling Arteriovenous malformation (AVMs). A combination of endothelial cells and fibroblasts were seeded in fibrin hydrogels for up to 7 days. **ii.** Day 7 images of mutant cell microvessels treated with MEK inhibitor starting at day 3 (scale bar 500 μm). Adapted from *Soon* et al. (2022) [123]. **F.** Four-month vascularised cerebral organoids were analysed for morphology by microscopy. H&E staining, and immunofluorescence analysis of the expression of pericyte marker alpha-SMA, vascular ECs markers vWF and CD31, and SOX2. Nuclei were stained with DAPI (blue). Scale bar, 200 μm. Adapted from *Ham* et al. (2020) [127]. SMA = smooth muscle actin; vWF = Von Willebrand factor **G.** Identification of vessel-like structures in the vascularised cerebral organoids maintained for up to 50 days. CD31^+^ = endothelial cells; SMA^+^ = smooth muscle cells; PDGFR^+^ = pericytes; ColIV^+^ = collagen IV; CD144^+^ and KDR^+^ = endothelial progenitor cells; TUJ1^+^ = neural networks Adapted from *Ahn* et al. (2021) [134].

To control cell structure and formation, immortalised cell lines have been used to create astrocyte and pericyte spheroids, covered in human brain endothelial cells after 2 days, which demonstrated selective permeability to glucose analogues and not to large molecular dextran [118]. To provide a third separate layer, *Cho* et al. generated spheroids of mostly astrocytes, surrounded by pericytes and then endothelial cells and evidenced transport and regulatory processes that are key to BBB function [119]. Together, culturing brain endothelial cells, astrocytes and pericytes in low-adherence plates self-assemble into BBB spheroids [120]. *Bergmann* et al. did not characterise the structure of the cells within the spheroids, and how they interacted with each other however, did determine that these models offer a valid platform for drug permeability assays, investigating rate of diffusion through the cell layers to the centre of the spheroid. These models are useful to provide insight into the cellular complexity of BBB and drug transcytosis, but difficult to perfuse and lack the cerebral architecture for ICH study. Exploiting the self-assembling ability of cells, *Goldman* et al. differentiated iPSCs to develop the iBBB model contained in Matrigel [121]. This platform was constructed to model cerebral amyloid angiopathy (CAA) by adding amyloid fibrils, which is a clinical risk factor for ICH. *Simonneau* et al. advanced this model using hydrogels to micropattern a 96 well plate and generated thousands of vessel organoids in a single plate [122]. Characterising the cell assembly within the organoid, astrocytes were located in the core being surrounded by pericytes, and endothelial cells generated the surface layer of the spheroid. Similarly, *Soon* et al. modelled arterio-venous malformations (AVM)s using mutant human endothelial cells to replicate vessels that are malformed and vulnerable to rupture, another neurological condition that carries risk for ICH [123] (Fig. 6). AVMs are notoriously difficult to generate in animal models and there is an urgent need for alternatives with accurate pathology representation. Furthermore, it was demonstrated that mutant cells formed vessels on a chip of fibrin gel and demonstrated typical disease pathology; vessels were permeable, enlarged and had decreased branching. Although brain cells were not included in the model, it presents a promising platform for exploring ICH risk.

Standard protocols for generating human vascular organoids have been developed, along with culture kits to standardise the process across researchers [124]. Human iPSCs can be cultured using specific media formations that contain the potent and selective aminopyrimidine derivative CHIR99021 for mesodermal differentiation and VEGF-A and forskolin to initiate vascular cell (endothelial cells and pericytes) differentiation (Fig. 6). The organoid produces a vessel network with realistic morphology and cell-cell relationships, endothelial cell networks wrapped in smooth muscle cells and pericytes, that deposit basement membrane proteins.

### 3.2 Vascularised 3D brain models and applications in ICH modelling

Although self-assembly of vascular cells in a 3D hydrogel forms the most physiologically relevant model of the cerebral vasculature, considering cell types and lumenised morphology, the lack of cerebral tissue limits their potential for ICH modelling. Since the advent of cerebral organoids in 2013 [125], there have been standardised protocols and commercial kits available to regulate their production, with even ready-made brain-region-specific organoids (see Human iPSC-Derived Midbrain Organoids provided by STEMCELL™ Technologies). Although cerebral organoids mimic heterogenous architecture, cell differentiation, and physiology of the developing human brain, they lack vascularity and therefore are limited in their potential for stroke research [126]. Optimising the vascularisation of cerebral organoids has presented itself as the next step, to enable flow of media through the tissue and allow for longer culture and relevant cell maturation. *Ham* et al. revealed that vascular differentiation can be initiated in cerebral organoids using vascular endothelial growth factor (VEGF) during embryoid formation without inhibiting neural differentiation [127] (Fig. 6). VEGF is essential for neuroprotection in the developing neural system, neuronal patterning and angiogenesis after stroke. Authors demonstrated that the vascular cells undergo angiogenic sprouting and spontaneously form blood vessels wrapped in pericytes, expressing vascular development genes. Co-culturing cerebral spheroids with endothelial cells in low-attachment plates resulted in the formation of ZO-1 positive tight junctions and pericytes, with spontaneous merging of the endothelial cells with the spheroids after 2 weeks [128] and investigated the potential to use ultrasound microbubbles to cross the BBB for drug delivery mechanisms. The incorporation of endothelial cells in cerebral organoid cultures accelerates the differentiation of cells and organisation of tissue, as brain-derived neurotrophic factor (BDNF) is released from endothelial cells [129].

*Sun* et al. generated vascular organoids with neurotrophic reagents N2 and B27 to induce brain vessel specific features such as the spontaneous co-differentiation of microglia [130]. These organoids were perfusable with microinjection and showed genetic and morphological similarities to *in vivo* analysis of the BBB. This was followed by fusing the brain specific vascular organoids with embryoid bodies that had undergone neural ectoderm induction, and by day 40 integrated vessel networks, BBB structures such as claudin-5 and ZO-1, were observed in the fused organoids. They also noted microglia throughout the 40-day tissue, differentiation of which is not spontaneous following the standard cerebral organoid differentiation protocols.

The integration of multiple region-specific brain organoids results in the formation of complex self-organizing *in vitro* constructs, termed assembloids [131,132]. These systems exhibit enhanced physiological relevance and enable the investigation of intricate model development and associated neurodevelopmental processes [133] - such as an alternative strategy to integrate vasculature, to investigate inter-regional cell migration, long-range axonal pathfinding, or establishment of functional neural circuits, that are challenging to be recapitulated by conventional single-region organoid models.

*Ahn* et al. generated human vascular organoids and cerebral organoids from standard protocols and attached the vascular plexus to the developing cerebral organoids forming assembloids on day 5, by embedding clumps of vascular organoid in Matrigel during the neural induction stage [134] (Fig. 6). Immunofluorescence revealed these cells have vessel-like structure until day 50, associating with smooth muscle cells and pericytes, integrated with the neural cells. Although previously demonstrated that vascular differentiation can occur within the developing cerebral organoid, *Ahn* et al. determined that these vascular networks were derived solely from the implanted nexuses. They did not observe astrocytes covering the endothelial cell network, perhaps because these cells were derived from different origins too early in development, or perhaps because there was no perfusion to recruit astrocytes to the vessel surface. Crosstalk between endothelial cells and neural stem cells promote both vascular and neural fates and determine the ECM composition [135]. *Dao* et al. utilised the cerebral organoid and blood vessel organoid assembloids to generate a model of cerebral cavernous malformations (CCMs), a condition similar to AVMs for which there are inadequate animal models [133]. Clinically CCMs are a risk factor for ICH, as the malformed vessels are often weakened by poor microstructure, thin walls and dilated lumens. Patient cells were used to create both cerebral and vascular organoids and exhibited pathology similar to resected human tissue through a loss of vascular smooth muscle cells.

### 3.3 Incorporation of microfluidics approaches to ICH organoid models

**Fig. 7:**
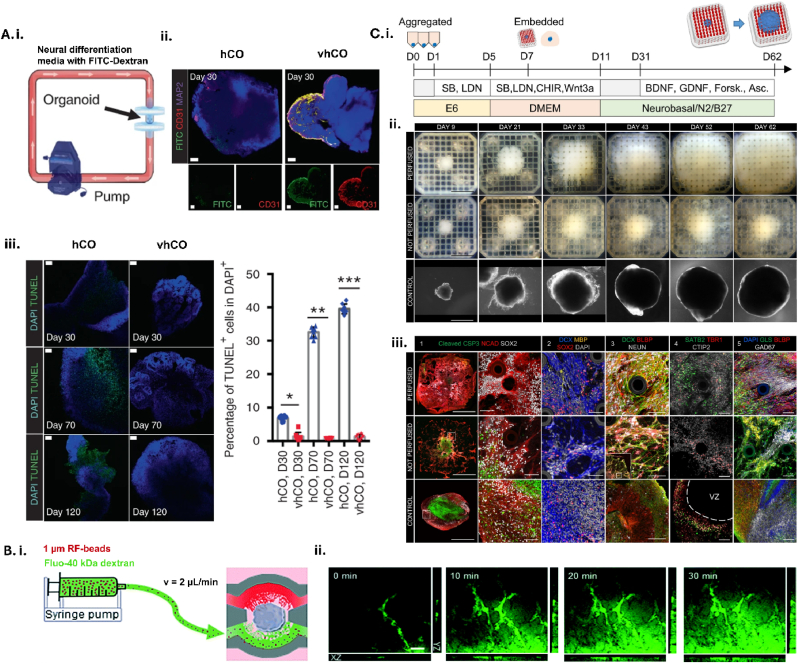
– **Perfusion of 3D culture systems to recreate a microphysiological system of the cerebrovasculature A. i.** Illustration of FITC-dextran perfusion into organoids via bioreactor with the flow rate of 0.88 ml/min **ii.** Immunostaining of whole mount FITC-dextran perfused vhCOs and control hCOs for CD31. **iii.** TUNEL staining of organoids and quantification of TUNEL^+^/DAPI ^+^ cells indicated that the increase in cell death at the center of control hCOs at day 70 and 120 was dramatically reduced in vhCOs. Adapted from *Cakir* et al. (2019) [136]. CD31 = endothelial cells; MAP2 = microtubule-associated protein 2; hCO = human cerebral organoids; vhCO = vascularised human cerebral organoids; TUNEL = apoptotic cells. **B. i.** Sketch of microfluidic chip perfusion experiments with syringe pump and fluorescent dextran **ii.** fluorescein 40 kDa perfusion at four different time points (scale: 100 μm). Adapted from *Salmon* et al. (2022) [137] **C. i.** Schematic representation of experimental and differentiation protocols for perfused human organoids with a synthetic vasculature. **ii.** Bright field images of organoids in perfused (top row), non-perfused (middle row) grids and control organoids (bottom row) **iii.** Immunofluorescent images of transverse sections of the three sample types. Adapted from *Grebenyuk* et al. (2023) [138]. NCAD = N-Cadherin; SOX2 = neural stem cells; MBP = myelin basic protein; DCX = doublecortin; BLBP = radial glia; NeuN = mature neurons; SATB2 = upper cortical layers; TBR1/CTIP2 = deep cortical layers; GAD67 = GABAergic neurons; VZ = ventricular zone.

In order to accurately mimic the human condition of blood rupture into these systems, perfusion is essential. However, as the models increase in complexity, and incorporate the heterogeneity of organoid formation, it becomes more challenging to generate reproducible and accurate model systems for investigation. Current state-of-the-art presents several methods to incorporate fluidic systems with the model platforms. *Cakir* et al. engineered embryonic stem cells to express ETS variant 2 (ETV2), a haematopoietic transcription factors for vascular development, and co-created vascular networks within the developing cerebral organoid that aided maturation and expressed critical BBB markers such as active transporter proteins and tight junctions [136] (Fig. 7). These organoids were perfused using dextran, held between two filters connected to a pump system to determine the lumenisation of the vessels, a step of vasculogenesis that occurs simultaneously with the onset of flow. *Salmon* et al. engineered a specialised chip that allowed vascular cells to flow across the organoid and create vessel networks through the embedding Matrigel [137] (Fig. 7). These vessels penetrated the organoid, creating pericyte layers and depositing ECM however, transition from a 2D layer of endothelial cells to a 3D organoid resulted in limited penetration of the cerebral tissue (∼40 μm). The vessel network was perfusable across the invaded microtissue, and by linking several chips together, this presents a scalable platform to investigate the addition of human blood into the media. Similarly, *Grebenyuk* et al. designed a microfluidic meshwork of synthetic vessels to perfuse cerebral organoids [138] (Fig. 7). They demonstrated that perfusion prevents internal tissue from becoming hypoxic, and there is a merging of cerebral organoids to make one large structure. Such a model would not be used to investigate the endothelial cell involvement however, could be vital to investigating the pathogenic effect of blood release into the cerebral tissue. Moreover, spontaneous vascular differentiation within a larger structure was not further investigated. Addition of VEGF to create an integrated network through the organoid tissue to initiate differentiation and angiogenesis would have been appealing.

## 4 Future applications for ICH modelling

The requirement for accurate, human ICH models has been a topic of discussion for some years [139]. Priorities for effective clinical translation of preclinical ICH research, as outlined by the Hemorrhagic Stroke Academia Industry (HEADS) roundtable [139] and inspired by the Stroke Therapy Academic Industry Roundtable (STAIR) guidelines for ischaemic stroke [140] include 1. developing models of more clinically relevant ICH (in size and occurrence), 2. to compare vascular pathophysiology in spontaneous models to human condition 3. to understand the impact of comorbidities in spontaneous ICH 4. determine the pathophysiology of genetic causes of ICH and 5. develop methods to rapidly detect spontaneous ICH in animals. To develop novel therapies, that are effective across both haemorrhagic and ischaemic stroke, it is vital to better understand the pathology that can be therapeutically targeted. As alternatives to the transwell systems, microfluidic systems present challenges that include researcher labour and user-variability limiting the translatability of the models. No standardisation in the appropriate control conditions, chip fabrication and design, materials and cell type, means there is a lack of reproducibility. In addition, commercially available chips are still user-attuned and therefore variable between applications. However, there is hope that progress is being made within a regulatory framework through the 2022 Food and Drug Administration (FDA) Modernization Act 2.0 and the recent 2024 European Committee for Standardization (CEN)/The European Committee for Electrotechnical Standardization (CENELEC) Organ-on-Chip Standardization Roadmap [141] which are game-changing milestones in the organ-on-chip landscape. Desirable high-fidelity models must be reproducible and accurate, but also scalable for high-throughput analysis, translatable to clinical care, and enable accessible vasculature to mimic therapeutic routes into the brain. It is therefore a combination of model platforms and incorporating multi-modal systems to mimic increasing disease complexity (e.g., microfluidic flow + organoid complexity) that will push the preclinical modelling capacities for translation and reduce the reliance on animals.

The potential for cerebral organoids in human neuroscience has been realised, addressing questions of neurodevelopment and precision techniques involving the formation of patient-derived organoids however, not devoid of limitations. Developing cerebral organoids do not form homogenous brain tissue and even using commercially available kits can generate tissue that form multiple rosettes of neuronal differentiation, significant fluid-filled spaces (i.e., choroid plexus) and different regions of cell specialisation. Although haemorrhages occur in different locations throughout the brain (lobar, cerebellum, basal ganglia) and this may be represented in a heterogenous tissue, there is little reproducibility with such variation. Characterisation of these microtissues is not standardised, and genetic origin of the stem cells and immortalised cell lines cause many differences during development. Some efforts have been made to generate a tool-kit for the standardisation of organoid characterisation [142] and ethical regulation across the organ-on-chip field however, are currently in their infancy. As organoids grow larger with perfusion, they are optically opaque and difficult to visualise internally without tissue processing. Visualising internal effects of perfusion and blood exposure in live, intact tissue presents the same limitations as animals. Although the wide use of Matrigel to model the ECM, there is a lack of macrostructure that also influences cell function and disease pathology such as directed neuronal connectivity, micro and macro vasculature, an encasing meninges/skull for mechanical pressure on the tissue, and the peripheral immune system. There have been a number of different culture methods to include microglial cells in the cerebral organoid tissue, however the full impact of these different cell origins and the impact on development has not yet been sufficiently characterised [143]. There are expenses associated with long term stem cell culture that render these >40-day long culture experiments not always widely accessible, or more economical than rodents. The field of cerebral organoids is relatively new and the state-of-the-art is advancing to address these limitations quickly. ICH is a multifactorial disease and breaking down the disease to composite parts and include the models above in pre-clinical study could be advantageous.

Another aspect of ICH modelling that needs to be addressed is the addition of blood to the culture. When adding blood to the 3D models of the BBB, blood introduces optical opacity and a mix of cell types that are difficult to trace. Some models only add hemin or haemoglobin in isolation to initiate the neurotoxicity however, without whole blood there is a whole complement of pathological factors that are missed. Thrombin, free iron, leukocytes and immune factors, platelets and oxidised free radicals are all neurotoxic causing neuroinflammation and cell death. Therapeutic options for ICH are to stop the bleeding, reduce the rebleeding and to surgically remove the heamatoma that often occurs with downstream vascular damage. Alternative options that may benefit from the above pre-clinical modelling include absorbing and neutralising the neurotoxic factors in blood within the parenchyma, protecting the neurons from inflammation and cellular death, and speeding up recovery by promoting haematoma clearance without surgery.

## 5 Conclusions

This review highlights on important and relevant models that can be used as pre-clinical ICH research platforms to address different questions about pathological aetiology, drug delivery and efficacy, and recovery. However, very few have been employed in such research direction, and the emphasis for pre-clinical ICH investigations remains on animal models. There are platforms to interrogate aspects of disease however, to recreate an accurate model of pathology, incorporation of a multitude of different cell types (vascular, neural and immune) is required in an open accessible system. This system should also contain a flow that can be manipulated and regulated with adequate, accurate controls if there is ever to be an upscale to drug discovery, or nanotechnological screening, for facilitated clinical translation and future use in patients.

## CRediT authorship contribution statement

**Siobhan Crilly:** Writing – original draft, Visualization, Methodology, Investigation, Funding acquisition, Conceptualization. **Mihai Lomora:** Writing – review & editing, Visualization, Supervision, Investigation, Funding acquisition, Conceptualization.

## Ethics approval and consent to participate

The authors declare that Ethics approval and consent to participate is not applicable for this manuscript.

## Declaration of competing interest

The authors declare that they have no known competing financial interests or personal relationships that could have appeared to influence the work reported in this paper.
